# *In Vitro* Culture and Characterization of a Mammary Epithelial Cell Line from Chinese Holstein Dairy Cow

**DOI:** 10.1371/journal.pone.0007636

**Published:** 2009-11-03

**Authors:** Han Hu, Jiaqi Wang, Dengpan Bu, Hongyang Wei, Linyun Zhou, Fadi Li, Juan J. Loor

**Affiliations:** 1 State Key Laboratory of Animal Nutrition, Institute of Animal Science, Chinese Academy of Agricultural Science, Beijing, China; 2 Faculty of Animal Science & Technology, Gansu Agricultural University, Lanzhou, China; 3 Mammalian NutriPhysioGenomics, Department of Animal Sciences and Division of Nutritional Sciences, University of Illinois, Urbana, Illinois, United States of America; Health Canada, Canada

## Abstract

**Background:**

The objective of this study was to establish a culture system and elucidate the unique characteristics of a bovine mammary epithelial cell line *in vitro*.

**Methodology:**

Mammary tissue from a three year old lactating dairy cow (ca. 100 d relative to parturition) was used as a source of the epithelial cell line, which was cultured in collagen-coated tissue culture dishes. Fibroblasts and epithelial cells successively grew and extended from the culturing mammary tissue at the third day. Pure epithelial cells were obtained by passages culture.

**Principal Findings:**

The strong positive immunostaining to cytokeratin 18 suggested that the resulting cell line exhibited the specific character of epithelial cells. Epithelial cells cultured in the presence of 10% FBS, supraphysiologic concentrations of insulin, and hydrocortisone maintained a normal diploid chromosome modal number of 2n = 60. Furthermore, they were capable of synthesizing β-casein (CSN2), acetyl-CoA carboxylase-α (ACACA) and butyrophilin (BTN1A1). An important finding was that frozen preservation in a mixture of 90% FBS and 10% DMSO did not influence the growth characteristics, chromosome number, or protein secretion of the isolated epithelial cell line.

**Conclusions:**

The obtained mammary epithelial cell line had normal morphology, growth characteristics, cytogenetic and secretory characteristics, thus, it might represent an useful tool for studying the function of Chinese Holstein dairy cows mammary epithelial cell (CMECs).

## Introduction

Mammary tissue cells or explants have been widely-used over the years as models to understand the physiological function of mammary gland. When using tissue explants, however, it is inherently difficult to distinguish between primary mitogens and secondary regulators of mammary gland function/development. To circumvent most of these difficulties, emphasis has been placed on cell culture methodologies to study growth regulation, hormonal responsiveness, or biochemical properties of mammary epithelial cells (MEC). Some of these previous work have led to the development of stable epithelial cell lines of bovine mammary gland [1]–[2].

A method for obtaining primary epithelial cells from human milk has been described [3]–[4]. Collagenase dissociation was used successfully during isolation and culture of bovine epithelial cells *in vitro* [5]–[6]. Tissue culture is another method which was successfully used to isolate bovine mammary epithelial cells [7]. Bovine mammary epithelial cell lines reported in the past include BMEC+H [8], MAC-T [1], PS-BME [5], BME-UV [9] and L-1 [10]. In these studies, the isolated cells were not immortal, thus, additional work was required to develop a bovine mammary epithelial cell line.

Collagen is a universal factor used for mammary epithelial cell cultures *in vitro*. Although intricate, two- and three-dimensional substrates for cell culture have been described, collagen is still one of the simplest and most commonly-used matrices [11]. In previous protocols, mammary epithelial cells were ordinarily cultured in DMEM-F12 (1∶1) medium supplemented with 10% FBS and several bioactive factors including insulin, growth hormone, hydrocortisone, and epidermal growth factors [10], [12]–[13]. Some of those bioactive factors can influence the rate of mammary gland involution as well as metabolism [14]–[15]. For example, Colomb [16] showed that estradiol and epidermal growth factor are required for cell-cycle progression of normal human mammary epithelial cells in culture. Hydrocortisone has a stronger effect on milk protein synthesis than on total protein synthesis [10]. Supraphysiological concentrations of insulin are required for optimal viability of epithelial cells [15]. A more thorough investigation of substrate requirement for cell growth in BME-UV mammary epithelial cells showed that the most important supplements were lactalbumin hydrolysate, hydrocortisone, and insulin because their omission reduced cell proliferation [17].

Currently, the Chinese Holstein cow is the main dairy cow breed in China. However, few reports dealing with mammary epithelial cell function of Chinese Holsteins have been published [18]. With the fast development of the Chinese dairy industry, more attention is being placed on the mechanisms or factors that might affect milk synthesis and quality. Thus, a primary objective of this investigation was to isolate and culture CMECs *in vitro* so that their potential as a model to study MEC function in this breed of cattle could be evaluated. The isolated CMECs were thoroughly characterized via morphology, chromosomal analysis, immunocytochemistry [9], [19], RT-PCR, and Western-Blotting analysis [13].

## Materials and Methods

### Ethics Statement

In the present experiment, animal care and procedures were approved and conducted under established standard of the Institute of Animal Science, Chinese Academy of Agricultural Sciences, Beijing, China.

### Materials

The basal growth media was DMEM/F12 containing 10% fetal bovine serum (FBS) (Invitrogen, Beijing Maojian United Stars Technology Co., Ltd., China). Induction media, which could promote the synthesis of milk protein and fat, was the growth media containing 5 µg/mL bovine insulin, 5 µg/mL bovine Holo-transferrin, 5 µg/mL progesterone, 10^−7^ mol/L hydrocortisone, 10 ng/mL bovine epithelial growth factor and 5 µg/mL bovine estradiol (Sigma-Aldrich, cat. #I4434, T1283, P8783, H0888, E4127, E2758, respectively). The storage media prepared freshly was composed of 90% fetal bovine serum and 10% DMSO. A solution of 0.25% trypsin-0.02% EDTA solution (Sigma-Aldrich) used for cell digestion was prepared and stored at −20°C until use.

### Tissue Isolation

Bovine mammary tissue was obtained from a three year old mid-lactation (ca.100 d relative to parturition) Chinese Holstein dairy cow. Fresh tissue was placed in sterilized tubes containing ice-cold D-Hank's (balanced salt solution) and immediately transported to the laboratory. Tissue of ca. 1 cm^3^ was washed with D-Hank's solution for several times until the solution was pellucid and without milk. Tissue was then cut into 0.5∼1 mm^3^ cubes and washed again with D-Hank's solution until clean. These smaller pieces of tissue were transferred with sterile tips onto empty plastic cell culture dishes (Corning, 430165,U.S.A) coated with collagen. Care was taken to ensure that tissue was kept wet. Culture dishes were incubated at 38°C and 5% CO_2_ and were monitored closely every 30 min. If the adjacent area surrounding the tissue was dry, several drops of basal media were added ensuring that the tissue would not float and separate from the bottom of the culture dish. After 4 h, 0.5 mL basal media were added to every culture dish and 1 mL basal media were added after 12 h. The basal media was replaced with fresh media every 48 h until cells were visibly spread across the bottom of the culture dish. Cells were detached with 0.25% trpysin-0.02% EDTA and transferred to new culture dishes, which were used to remove fibroblasts. Subsequently, the pure mammary epithelial cells were isolated after 3 passages.

### Growth Characteristics of Epithelial Cells

Growth curves and doubling time were determined by seeding 5×10^4^ cells/well in 12-well flat-bottom culture plates (Corning 3513, U.S.A) containing induction media. Cell number and viability were determined each day in triplicate wells between 7 to 11 d post-seeding via trypan blue exclusion. Morphology of cultured cells was routinely evaluated with an inverted microscope with phase contrast (Olympus IX71, Japan), and photomicrographs were taken.

### Karyotyping Analysis of Epithelial Cells

The cells from three periods (primary, purified, and resuscitated cells) were examined via changes in karyotyping analysis as described by Seabright [20]. Exponentially-growing cells were incubated with colchicine (0.2 µg/mL) for 2–2.5 h. Cells were trypsynized with 0.25% trypsin and treated with warm hypotonic KCl solution (0.075 mol/mL) for 30 min at 37°C. The solution was centrifuged at 1200 × g for 10 min and the cells were harvested. Cells were then fixed with ice-cold methanol and acetic acid mixture (volume 3∶1) 3×, commencing after 30 min and subsequently twice at 15 min intervals. Each time, cells were centrifuged at 1200 × g for 10 min prior to harvesting of cells. Cells were suspended with 0.5 mL fixed solution and spotted onto ice-cold glass slides. The sample slides were allowed to dry at room temperature and stored at −20°C until use. All the slides were stained with Giemsa solution (1.0 g giemsa, glycerin 66 mL,and methanol 66 mL) 1 mL/slide for 10 min, washed with distilled water and dried at room temperature. Chromosomes were visualized and detected with a phase-contrast microscope (Olympus IX71, Japan) and analyzed with the soft Video TesT Karyo3.1 (NatureGene Corp., USA).

### Immunocytochemistry

Cytomatrix and expression of cytokeratin 18 (abcam, ab668; UK) and vimentin (abcam, ab8978; UK) were examined by seeding 5×10^4^ cells/well in 12-well flat-bottom culture plates. Cytostructural protein expression was examined at 6 d after seeding cells that were cultured in induction media. Before staining, the cells were washed 3× with D'-Hanks solution and fixed with ice-cold methanol. Cells were incubated in PBS containing 50 µg/mL pronase (Sigma) at 37°C for 15 min, then rinsed with running tap-water. Cells were pre-incubated with 0.1% (w/v) phenylhydrazine-HCl (Sigma) for 5 min to inhibit endogenous peroxidases and washed with PBS. Nonspecific reactivity was blocked with poultry serum (Beijing CCpioneer Technology Co.,Ltd, China) for 1 h at room temperature. First antisera, anti-cytokeratin 18 and anti-vimentin were diluted 1/200 in PBS and incubated for 1 h at room temperature. The cells were washed 2× 5 min with PBST and once 5 min with PBS. Secondary antibody, FITC-conjugated monoclonal anti-mouse IgG (Sigma, F4143), was diluted 1/64 in PBS and incubated in the dark for 0.5∼1 h. Cells were washed for 3× with PBS and visualized with a phase-contrast microscope (Olympus IX71, Japan).

### RT-PCR

Total RNA from mammary tissue, purified, and resuscitated cells cultured with induction media was isolated with ice-cold TriZol solution (Invitrogen). The expression of CSN2, ACACA, and BTN1A1 were determined by RT-PCR. The integrity and concentration of the RNA were verified by analyzing 5 µL of each sample on a 1% agarose gel and ultraviolet spectrophotometer (Beckman DU800, U.S.A). Reverse transcription system (SuperScript^@^) was purchased from Invitrogen. Primers of CSN2, ACACA and BTN1A1 were designed with Primer 5.0 (table 1) and synthesized by Shanghai Sangon Biological Engineering Technology & Services Co. Ltd (China).

**Table 1:** Primer sequence and reactive condition of PCR.

Gene	GenBank Number	Primer sequence (F)	Primer sequence (R)	Reactive condition of PCR	Length (bp)
ACACA	NM_174224	5′-TCAGGGACTGCCGAAACATT	3′-TCAGGGACTGCCGAAACATT	94°C 4 min;94°C 30 s, 52°C 30 s, 72°C 1 min, 35cycles; 72°C 7 min	144
BTN1A1	Z93323	5′-TGTGTTGCTGCTGATAGAGTGTTAG	3′-CCTCCAAGTTCCTTTATGGGATTTC	94°C 4 min;94°C 30 s, 52°C 30 s, 72°C 1 min, 35cycles; 72°C 7 min	305
CSN2	S67277	5′-AGGAACAGCAGCAAACAG	3′-R5TTTCCAGTCGCAGTCAAT	94°C 4 min;94°C 30 s, 56°C 30 s, 72°C 1 min, 35cycles; 72°C 7 min	579

### Western-Blotting

Protein lysates from mammary tissue, purified cells, and resuscitated cells were prepared with TriZol solution (Invitrogen) and subjected to SDS-PAGE on 15% polyacrylamide gels. The resolved proteins were blotted onto PVDF transfer membranes (0.45 µM immobilon-P, Millipore, K6KN8780E) and blocked overnight with 3% poultry serum in TBST. Membranes were incubated with primary antibody at 4°C overnight, appropriately diluted 1∶4000 in TBST, washed with TBST 3×, and then incubated with HRP-conjugated secondary antibody diluted 1∶5000 in TBST. The polyclonal antibody of β-casein was prepared by JingMei Biotech Co. Ltd (China).

## Results

### Establishment of Bovine Mammary Gland Epithelial Cell Line

**Figure 1:**
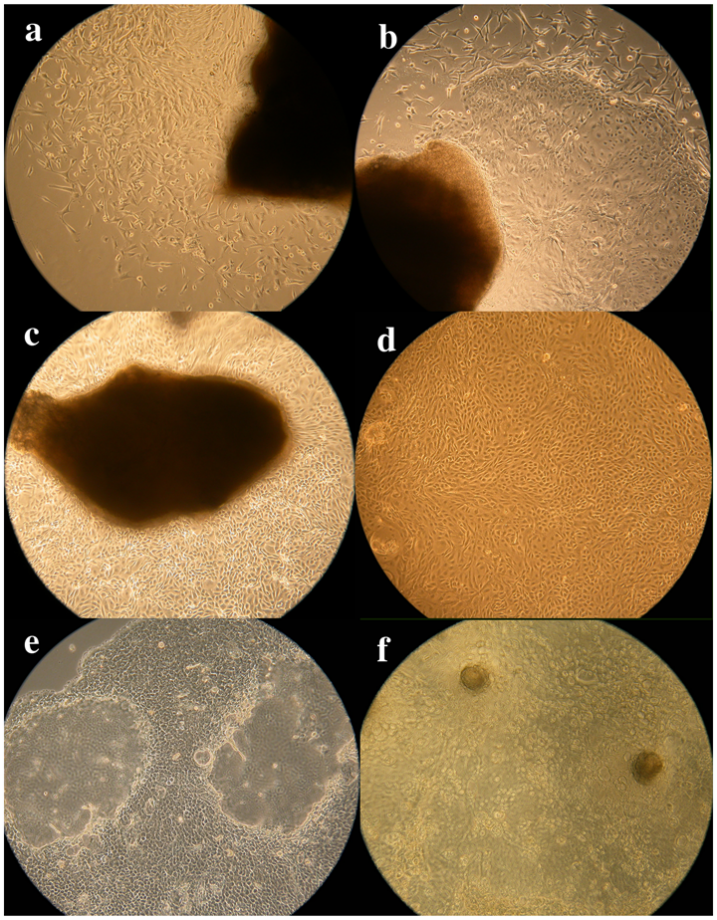
Photomicrographs of the process obtained bovine epithelial cells and morphology of epithelial cells. a. Fibroblast cells firstly grew from mammary tissue (×100) b. Synbiosis of fibroblast and epithelial cells (×100) c. Purified epithelial cells grew from mammary tissue (×100) d. Purified epithelial cells obtained by digestion (×100) e. Epithelial cells form dome-like structure (×100). f. Epithelial cells form papillate structure (×40).

The whole growth process of isolating CBMCs is exhibited in Fig. 1, including tissue culture and different stages of culturing epithelial cells. Primary fusiform fibroblasts were first elongated from mammary tissue after culture for 3 to 5 d (Fig. 1a). Fibrilar components grew flat and extended through the whole plate. Primary epithelial cells were elongated from mammary tissue after a week to 10 d in culture. Subsequently, these fibrilar cells died rapidly and numbers decreased. At the same time, epithelial cells began to proliferate and accumulated with fibrilar cells being located at the boundary of these two kinds of cells (Fig. 1b). After ca. two weeks, primary epithelial cells became the dominant cell type in plates (Fig. 1c). Generally, it took 10 to 15 min to detach the epithelial cells from culture dishes with trypsin and EDTA solution. However, less than 1 to 2 min was needed for fibroblasts. Thus, epithelial cells were isolated to homogeneity after 3 passages (i.e., trypsin digestion). The resulting bovine mammary epithelial cells were present as a cobblestone form and proliferating monolayer (Fig. 1d).

When the tissue was cultured in basal media, the epithelial cells extended from the tissue regardless of the number of times the tissue was digested with trypsin and EDTA. It was noteworthy that epithelial cells, not fibroblasts, grew continuously and extended even from the first tissue samples (Fig. 1c). However, the tissue was fragile and exfoliated easily from the plate. Thus, the degree of digestion and culture conditions seemed to be important for cell development. Mammary epithelial cells developed into different shapes. Most of the isolated cells that extended from the tissue had a cobble–stone-like shape, were connected tightly, and had a clear boundary (Fig. 1d). There were 2–4 nucleoli in each cell that were very distinct under the microscope. Under normal conditions and after ca. 10 passages, the volume of epithelial cells became larger and resembled big round flat fusiform shapes.

Cells obtained after freezing and thawing had normal morphology when re-cultured (Fig. 1d). In the process of epithelial cell culture, a dome-like structure (Fig. 1e) and mastoid process (Fig. 1f ) were evident, resembling the results of Pantschenko et al. and Heegard et al. [19], [21].

### Growth Characteristics of Epithelial Cells

**Figure 2:**
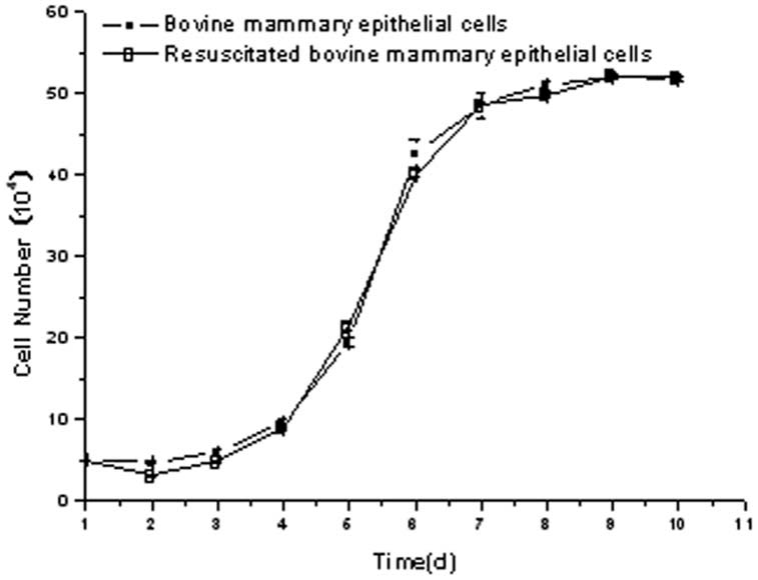
Growth curves of passage and resuscitated bovine mammary epithelial cells.

Growth curves showed that proliferation of BMECs (Fig. 2) whether from mammary gland tissue or those resuscitated doubled in number within 72 h when grown on plastic cell culture dishes and media with 10% FBS. The population of cells grew faster from d 4 to 6. When extended around the plastic substratum after d 8, the number did not change obviously in the last four days. Dead cells were observed floating in the media. These results are similar to those observed previously by Pantschenko [19], Zavizion [9] and Rose [6].

### Chromosomal Analysis of Epithelial Cells

**Figure 3:**
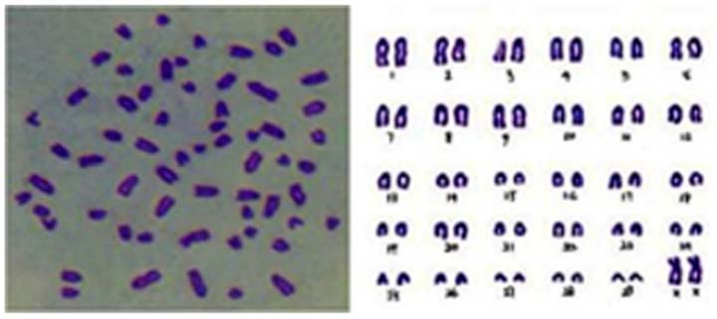
Karyotype of the chromosome of the obtained bovine mammary epithelial cells.

Chromosomal analysis demonstrated a non-transformed normal mammary epithelial cell lineage (Fig. 3). The isolated primary epithelial cells had a normal diploid configuration containing 60 chromosomes. Chromosomal analysis of CMECs before and after freezing also showed a modal number of 60.

### Cytoskeleton 18 and Vimentin Protein Expression

**Figure 4:**
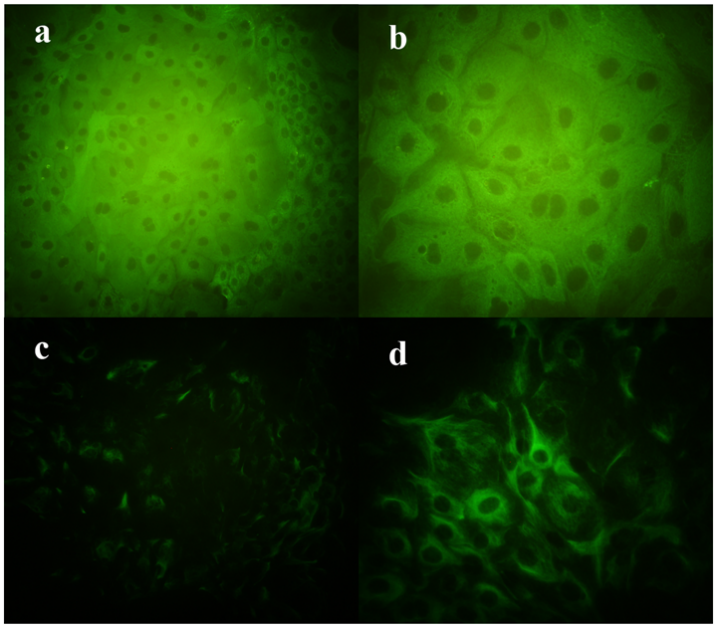
Immunocytochemistry of bovine mammary epithelial cells. Fluorescent image of cells incubated with anti-cytokeratin 18 monoclonal antibody (a×200, b×400). Fluorescent image of cells incubated with anti-Vimentin monoclonal antibody (c×200, d×400).

The expression of cytoskeleton and vimentin is directly consistent with the epithelial cell lineage [12], [19], [22]. Although the established cell line appeared to have epithelial cell morphology, we further investigated homogeneity at the eighth passage and also at the resuscitated cells by examining the protein expression of cytoskeleton 18, which is specific for epithelial cells, and vimentin which is usually expressed in various non-epithelial cells [23]. Both isolated and resuscitated epithelial cells exhibited intense positive staining of the cytoplasmic meshwork of cytokeratin fibrils when incubated with anti-cytoskeleton18 monoclonal antibody (Fig. 4a,b). Although nearly all the isolated and resuscitated epithelial cells in our study were positively-stained with anti-vimentin antibody (Fig. 4c,d), there was significant difference between the character of anti-cytoskeketon18 and anti-vimentin. The criteria for the positive staining reaction relied on structure as well as intensity. The wave filaments for cytokeratin revealed tonofilament junctions between cells which are important for intercellular communication and cellular polarity. Light stain pattern of vimentin was predominantly perinuclear with some evidence of filament degradation. The phenomena were completely consist with the results obtained by Pantschenko [19].

### Identification Epithelial Cells via Marker Proteins

**Figure 5:**
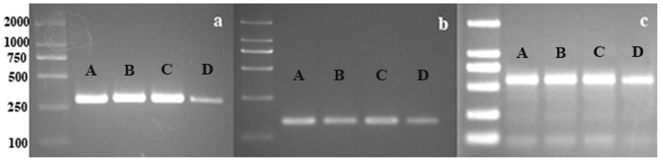
RT-PCR analysis of the amont of BTN1A1 (a), ACACA(b) and CSN2 (c). A: mammary gland; B: epithelial cells cultured in inducentment meida; C: resuscitated epithelial cells cultured in inducement meida; D: epithelial cells cultured in growth meida.

**Figure 6:**
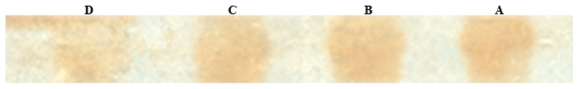
Western-Blotting analysis of the amont of β-casein. A: mammary gland; B: epithelial cells cultured in inducentment meida; C: resuscitated epithelial cells cultured in inducement meida; D: epithelial cells cultured in growth meida.

Establishing optimal culture conditions to allow for protein synthesis in mammary epithelial cells is of importance, as it would more closely mimic the *in vivo* system. Previous studies described testing β-casein expression in cultured epithelial cells [1]. Thus, such an approach was considered important in order to test the isolated cell system. We evaluated the protein synthesis ability of the isolated cells through mRNA and protein expression of CSN2, ACACA and BTN1A1. The total RNA and protein were isolated from native mammary tissue, isolated epithelial cells, epithelial cells cultured in induction media, and also in resuscitated epithelial cells. Expression of mRNA was determined by RT-PCR (Fig. 5). β-casein protein expression (Fig. 6) was detected by Western-Blotting as in previous reports [1]. The RT-PCR and Western-Blotting analysis results both confirmed the ability of the isolated cells to synthesize mammary-specific proteins. Furthermore, data showed that the induction media was able to enhance the ability of the isolated epithelial cells to synthesize protein *in vitro*.

## Discussion

In previous studies it was found that the concentration of fetal bovine serum (FBS) in media had a strong effect on the proliferation of bovine mammary epithelial cells. When compared with 0% FBS treatment, bovine mammary epithelial cells grown in 5 to 10% serum underwent a 3-to 4-fold increase in cell number during the 12 d of culture [11]. Cloned bovine mammary epithelial cells also depended on FBS for proliferation and a marginal advantage could be attributed to the higher FBS concentration (10% FBS) [10]. In the early stages of establishment of our system (data not shown), we examined the effect of different media and various FBS concentrations on the proliferation of epithelial cells. Effects were assessed by attachment rates, which can reflect the difference in proliferation among cell types [24]. The results suggested that DMEM and DMEM/F12 were more suitable than F12 and RPMI1640. Evaluation of the cell population doubling time suggested that 10% FBS was the optimal concentration in growth media. These results were consistent with previous reports [10]–[11], [25]. However, a frozen solution with 90% FBS and 10% DMSO was optimal for cell preservation. Results of the resuscitated cell population doubling-time showed that a greater degree of normal bovine mammary epithelial cells survived when cells were preserved frozen in FBS serum containing 10% DMSO than in growth media with 10% FBS and 10% DMSO (data not shown). For subsequent studies, our bovine primary mammary epithelial cells were cultured in DMEM/F12 with 10% FBS as growth media, and FBS serum with 10% DMSO were used for frozen cell preservation.

To determine the effect of frozen preservation on isolated bovine mammary epithelial cells, we cultured both isolated and resuscitated cells in induction media to test for population proliferation. Compared with previous studies showing a doubling of cell proliferation between 24–36 h of culture [10], [26], the latent phase of the isolated primary epithelial cells in our study was >72 h. A possible reason for this effect is that the cells obtained in our study were not of clonal lineage, as in previous studies [5]–[6]. Growth curves showed that there was no significant difference in proliferation between the cells before and after frozen reservation. During the first 3 d of the latent phase, the growth rate was slow but during the following 3 d of growth there was an increase in cell number followed by a steady phase for the last several days of culture. Thus, the growth curve conformed to the rule of “S” sigmoid curve for proliferating cells with a lag-phase, exponential phase, and steady phase [27]. This suggested that the isolated bovine mammary epithelial cells maintained a favorable growth performance after frozen preservation. Thus, frozen preservation did not affect the proliferation of our isolated bovine mammary epithelial cell line and this response was similar to the results of Cifrian [28]. McGrath [11] also suggested that mammary epithelial cells could be stored by frozen preservation without affecting the viability of the frozen cells upon re-culturing.

Vimentin is the intermediate filament protein normally expressed by cells of mesenchymal origin. Vimentin is considered as a marker of myoepithelial cells [12]. However, cytokeratin 18 is one of the most common members of the intermediate filament gene family, and generally exists together with its filament partner keratin 8. It is expressed in single layer epithelial tissues of the body and is specific for epithelial cells [19]. Immunocytochemical staining of goat mammary tissue showed that vimentin was present in myoepithelial cells but not in epithelial cells. However, cytokeratin 18 was found in both epithelial and myoepithelial cells of the goat mammary gland [22]. Bovine myoepithelial cells were positive to anti-vimentin and negative to anti-cytokeratin 18 monoclonal antibody [17].

In a previous report, few of the resulting primary bovine mammary epithelial cells with characteristic fibroblast morphology were positively-stained with anti-vimentin [13]. The different result is that vimentin was also seen in lactating bovine mammary gland epithelial cells grown without hormones; whereas, cultures grown in the presence of hormones expressed only cytokeratins, which are specific for epithelial cells [29]. Rose [6] found monolayers of bovine mammary epithelial cells were stained positive for anti-pan-cytokeratin, anti-type VII cytokeratin than for vimentin. They suggested that the epithelial cells grown on plastic plates had some characteristics of myoepithelial cells for weak positive staining to vimentin. In our study, the obtained bovine mammary epithelial cells, which had staining positive for both monoclonal anti-cytokeratin 18 and anti-vimentin, exhibited the same phenomenon as reported previously [23]. The positive staining for cytokeratin-18 was a powerful result to prove the specific epithelium character of CMECs. The phenomenon of CMECs to vimentin was possibly associated with the culture conditions, plastic dish, media, monolayer overspread, and growth without the presence of other cells.

The major milk protein genes are defined as mammary-specific and developmentally-regulated expressed genes. As such, they represent markers of mammary differentiation [30]. Epithelial differentiation is characterized by expression of milk proteins, such as β-casein and whey acidic protein, the production of milk fats rich in triglycerides, sources of energy, and essential fatty acids [31]. Casein secretion is the hallmark of the bovine mammary epithelial cells [30]. Acetyl-coa carboxylase (ACACA) plays a pivotal role in the regulation of fatty acid (FA) metabolism, which mediates the first committed step for incorporation of acetate carbon into FA [32]. Thereinto, ACACA, a cytosolic protein, provides cytoplasmic malonyl-CoA for FA synthesis, which is rate-limiting for the synthesis of long-chain fatty acids de novo. The enzyme is active in the lactating mammary gland and its activity level is affected by dietary and hormonal states of the animal [33]–[34]. Butyrophilin (BTN1A1), a major milk-fat-globule transmembrane glycoprotein, is also a mammary-specific protein in milk-fat secretion expressed on the apical surface of the mammary epithelial cells in the final stage of pregnancy and during lactation [35]–[37]. And its mRNA could not be detected in bovine heart, intestine, kidney, liver, ovary, or uterus [38]. Although the function of BTN1A1 is not fully understood, its expression profile suggests an important role in lactation [39].

There was no marked difference of ACACA transcript level between mammary gland tissue, isolated epithelial cell cultured in induction media, or resuscitated epithelial cells. However, the transcript level in isolated epithelial cells cultured in basal media was evidently lower. A possible reason could be that there was less substrate in media provided for FA synthesis, thus, the addition of hormone to the cell culture provided was insufficient to increase flux through the pathway. In previous studies, Beswick [40] analysed the ACACA mRNA and protein abundance in the mammary gland of Holstein cows receiving either bovine growth hormone or bovine growth hormone-releasing factor, and revealed that there was no significant influence of the hormone alone. However, the transcription level of BTN1A1 and CSN2 suggested that bioactive factors including hormone in induction media could promote upregulation of secretory protin genes. It is clear that insulin is not only essential for milk protein gene expression, but also stimulates and regulates milk protein synthesis at multiple levels in bovine mammary tissue [41]. Insulin is essential for accumulation of casein mRNA in mouse mammary epithelial cells [15] and bovine lactating mammary gland cultured *in vitro* [42]. Aoki [43] also showed that stage-specific mRNA expression of milk fat globule membrance glycoproteins including BTN1A1 in mouse mammary epithelial cells was regulated in a similar mechanism to that of CSN2. Our results indicated that the isolated and resuscitated mammary epithelial cells all had a normal secretory function.

Casein protein was found neither in the fetal calf serum nor in the medium, thus, the presence of this product in medium was useful as an indication of functional differentiation of mammary epithelial cells [1]. Greater amount of casein protein was accompanied by an increase in mRNA and expression of protein. It has been clearly shown that insulin is absolutely required for maximal synthesis of protein, and a nearly maximal effect was achieved with 50 ng/mL in explants cultured for 4d [42]. Thus, insulin has been consistently used to supplement culture medium of mammary epithelial cell lines. β-casein production in cells grown for 1,3,5,7 and 10d increased over time and was consistently associated with the dome-like structures [19]. The production of β-casein also reflected the state of epithelial cells cultured *in vitro*. Different cell lines have distinct abilities to secrete milk protein. For example, in MAC-T cells it was estimated that α-casein and ε-casein secretion was 50 ng of α-casein/mL of medium/24 h [17]. MAC-T cells normally produce casein at levels comparable to those of BME-UV cells [9]. Similarly, clonal and parental lines of MAC-T cells all produced β-casein but production by clonal cells was much lower and averaged 0.1 to 0.3 µg/mL per 24 h [26]. Our results that β-casein protein detected in epithelial cells cultured in induction medium also identified the function of CMBCs. This result was as the same as that of mRNA test. So we suggested that bioactive factors in induction media were potential contributor to milk-protein synthesis [10], [42].

This study demonstrated the establishment of a functional bovine mammary epithelial cell line from Chinese Holstein cattle, which exhibited normal extracellular matrix and was physiologically-responsive to hormones. This cell culture model can be applied in future investigations of lactation in this breed of cattle or in comparative studies of mammary function across cattle breeds.

## References

[1] Huynh HT, Robitaille G, Turner JD (1991). Establishment of bovine mammary epithelial cells (MAC-T): an in vitro model for bovine lactation.. Exp Cell Res.

[2] Zavizion B, Politis I, Gorewit RC (1992). Bovine mammary myoepithelial cells. 1. Isolation, culture, and characterization.. J Dairy Sci.

[3] Taylor-Papadimitriou J, Shearer M, Tilly R (1977). Some properties of cells cultured from early-lactation human milk.. J Natl Cancer Inst.

[4] Ceriani RL, Taylor-Papadimitriou J, Peterson JA, Brown P (1979). Characterization of cells cultured from early lactation milks.. In Vitro.

[5] Gibson CA, Vega JR, Baumrucker CR, Oakley CS, Welsch CW (1991). Establishment and characterization of bovine mammary epithelial cell lines.. In Vitro Cell Dev Biol.

[6] Rose MT, Aso H, Yonekura S, Komatsu T, Hagino A (2002). In vitro differentiation of a cloned bovine mammary epithelial cell.. J Dairy Res.

[7] Du J, Di HS, Wang GL (2007). Establishment of a bovine epithelial mammary cell line and its ultrastructural changes when exposed to heat stress.. Sheng Wu Gong Cheng Xue Bao.

[8] Schmid E, Franke WW, Grund C, Schiller DL, Kolb H (1983). An epithelial cell line with elongated myoid morphology derived from bovine mammary gland. Expression of cytokeratins and desmosomal plaque proteins in unusual arrays.. Exp Cell Res.

[9] Zavizion B, van Duffelen M, Schaeffer W, Politis I (1996b). Establishment and characterization of a bovine mammary myoepithelial cell line.. In Vitro Cell Dev Biol Anim.

[10] German T, Barash I (2002). Characterization of an epithelial cell line from bovine mammary gland.. In Vitro Cell Dev Biol Anim.

[11] McGrath MF (1987). A novel system for mammary epithelial cell culture.. J Dairy Sci.

[12] Ehmann UK, DeVries JT, Chen MS, Adamos AA, Guzman RC (2003). An in vitro model of epithelial cell growth stimulation in the rodent mammary gland.. Cell Prolif.

[13] Matitashvili E, Bauman DE (1999). Culture of primary bovine mammary epithelial cells.. In Vitro Cell Dev Biol Anim.

[14] Rosfjord EC, Dickson RB (1999). Growth factors, apoptosis, and survival of mammary epithelial cells.. J Mammary Gland Biol Neoplasia.

[15] Bolander FF, Nicholas KR, Van Wyk JJ, Topper YJ (1981). Insulin is essential for accumulation of casein mRNA in mouse mammary epithelial cells.. Proc Natl Acad Sci U S A.

[16] Colomb E, Berthon P, Dussert C, Calvo F, Martin PM (1991). Estradiol and EGF requirements for cell-cycle progression of normal human mammary epithelial cells in culture.. Int J Cancer.

[17] Zavizion B, van Duffelen M, Schaeffer W, Politis I (1996a). Establishment and characterization of a bovine mammary epithelial cell line with unique properties.. In Vitro Cell Dev Biol Anim.

[18] Li JX, Zhang Y, Ma LB, Sun JH, Yin BY (2009). Isolation and culture of bovine mammary epithelial stem cells.. J Vet Med Sci.

[19] Pantschenko AG, Woodcock-Mitchell J, Bushmich SL, Yang TJ (2000a). Establishment and characterization of a caprine mammary epithelial cell line (CMEC).. In Vitro Cell Dev Biol Anim.

[20] Seabright M (1971). A rapid banding technique for human chromosomes.. Lancet.

[21] Heegard CE, White JH, Zavizion B, Turner JD, Politis I (1994). Production of various forms of plasminogen activator and plasminogen activator inhibitor by cultured mammary epithelial cells.. J Dairy Sci.

[22] Li P, Wilde CJ, Finch LM, Fernig DG, Rudland PS (1999). Identification of cell types in the developing goat mammary gland.. Histochem J.

[23] Pantschenko AG, Barber MR, Woodcock-Mitchell J, Bushmich SL, Yang TJ (2000b). Establishment and characterization of a caprine mammary myoepithelial cell line (CMMyoEC).. In Vitro Cell Dev Biol Anim.

[24] Vogler EA, Bussian RW (1987). Short-term cell-attachment rates: A surface-sensitives test of cell-substrate compatibility.. Journal of Biomedical materials research.

[25] Shamay A, Gertler A (1986). A model for in vitro proliferation of undifferentiated bovine mammary epithelial cells.. Cell Biol Int Rep.

[26] Zavizion B, Gorewit RC, Politis I (1995). Subcloning the MAC-T bovine mammary epithelial cell line: morphology, growth properties, and cytogenetic analysis of clonal cells.. J Dairy Sci.

[27] BAAS BECKIN LGM (1945). On the analysis of sigmoid curves.. Analysis of sigmoid curves.

[28] Cifrian E, Guidry AJ, O'Brien CN, Keys JE, Marquardt WW (1994). Bovine mammary teat and ductal epithelial cell cultures.. Am J Vet Res.

[29] Sommers CL, Walker-Jones D, Heckford SE, Worland P, Valverius E (1989). Vimentin rather than keratin expression in some hormone-independent breast cancer cell lines and in oncogene-transformed mammary epithelial cells.. Cancer Res.

[30] Fox PF, McSWEENEY PLH (2006). Advanced diary chemistry Volume 1: Proteins. Third Edition..

[31] Aoki N (2006). Regulation and functional relevance of milk fat globules and their components in the mammary gland.. Biosci Biotechnol Biochem.

[32] Fox PF, McSweeney PLH (2006). Advanced dairy chemistry Volume 2 Lipid, Third Edition..

[33] Mao J, Marcos S, Davis SK, Burzlaff J, Seyfert HM (2001). Genomic distribution of three promoters of the bovine gene encoding acetyl-CoA carboxylase alpha and evidence that the nutritionally regulated promoter I contains a repressive element different from that in rat.. Biochem J.

[34] Abu-Elheiga L, Brinkley WR, Zhong L, Chirala SS, Woldegiorgis G (2000). The subcellular localization of acetyl-CoA carboxylase 2.. Proc Natl Acad Sci U S A.

[35] Banghart LR, Chamberlain CW, Velarde J, Korobko IV, Ogg SL (1998). Butyrophilin is expressed in mammary epithelial cells from a single-sized messenger RNA as a type I membrane glycoprotein.. J Biol Chem.

[36] Mather IH, Jack LJ (1993). A review of the molecular and cellular biology of butyrophilin, the major protein of bovine milk fat globule membrane.. J Dairy Sci.

[37] Szyda J, Komisarek J (2007). Statistical modeling of candidate gene effects on milk production traits in dairy cattle.. J Dairy Sci.

[38] Jack LJ, Mather IH (1990). Cloning and analysis of cDNA encoding bovine butyrophilin, an apical glycoprotein expressed in mammary tissue and secreted in association with the milk-fat globule membrane during lactation.. J Biol Chem.

[39] Szyda J, Komisarek J (2007). Statistical modeling of candidate gene effects on milk production traits in dairy cattle.. J Dairy Sci.

[40] Beswick NS, Kennelly JJ (1998). The influence of bovine growth hormone and growth hormone releasing factor on acetyl-CoA carboxylase and fatty acid synthase in primiparous Holstein cows.. Comp Biochem Physiol C Pharmacol Toxicol Endocrinol.

[41] Menzies KK, Lefevre C, Macmillan KL, Nicholas KR (2009). Insulin regulates milk protein synthesis at multiple levels in the bovine mammary gland.. Funct Integr Genomics.

[42] Gertler A, Weil A, Cohen N (1982). Hormonal control of casein synthesis in organ culture of the bovine lactating mammary gland.. J Dairy Res.

[43] Aoki N, Ishii T, Ohira S, Yamaguchi Y, Negi M (1997). Stage specific expression of milk fat globule membrane glycoproteins in mouse mammary gland: comparison of MFG-E8, butyrophilin, and CD36 with a major milk protein, beta-casein.. Biochim Biophys Acta.

